# Prevalence and Characterization of Oxacillin Susceptible *mecA*-Positive Clinical Isolates of *Staphylococcus aureus* Causing Bovine Mastitis in India

**DOI:** 10.1371/journal.pone.0162256

**Published:** 2016-09-07

**Authors:** Hiral Mistry, Paresh Sharma, Sudipta Mahato, R. Saravanan, P. Anand Kumar, Vasundhra Bhandari

**Affiliations:** 1 National Institute of Animal Biotechnology-DBT, Hyderabad, India; 2 Department of Animal Genetics and Breeding, Veterinary College and Research Institute, Namakkal, Tamil Nadu, India; 3 Department of Veterinary Microbiology, NTR College of Veterinary Science, SVVU, GANNAVARAM, Andhra Pradesh, India; Rockefeller University, UNITED STATES

## Abstract

Bovine mastitis caused by multidrug resistant *Staphylococcus aureus* is a huge problem reported worldwide, resulting in prolonged antibiotic treatment and death of livestock. The current study is focused on surveillance of antibiotic susceptibility along with genotypic and phenotypic characterization of the pathogenic *S*. *aureus* strains causing mastitis in India. One hundred and sixty seven milk samples were collected from mastitis-affected cows from different farms in India resulting in thirty nine isolated *S*. *aureus* strains. Antibiotic sensitivity profiling revealed the majority of the strains (n = 24) to be multidrug resistant and eleven strains showed reduced susceptibility to vancomycin (MICs = 2μg/ml). All strains were oxacillin sensitive, but 19 strains were positive for the *mecA* gene, which revealed the occurrence of oxacillin susceptible *mecA* positive strains (OS-MRSA) for the first time from India. Additionally, 32 strains were positive for the *pvl* gene, a virulence determinant; of these 17 were also OS-MRSA strains. Molecular characterization based on multilocus sequence typing (MLST), *spa* typing, *agr* typing and SCC*mec* classification revealed strains belonging to different groups. Moreover, strains showed *spa* types (t2526, t9602) and MLST sequence types, ST-72, ST-88 and ST-239 which have been earlier reported in human infections. The prevalence of OS-MRSA strains indicates the importance of including both the genetic and phenotypic tests in characterizing *S*. *aureus* strains. Increased genotypic variability with strain related to human infections and *pvl* positive isolates indicates a worrisome situation with the possibility of bilateral transfer.

## Introduction

*Staphylococcus aureus* causes numerous infections in humans including sepsis, toxic shock syndrome, bacteremia, endocarditis, and surgical wound infections while in livestock animals bovine mastitis is the mainly reported disease [1]. India is the second largest milk producer in the world and bovine mastitis infections result in huge economic loss from decreased milk yield and poor animal health [2–4]. *S*. *aureus* is a major pathogen causing mastitis, and different states of India report a high prevalence rate of 57% and 41.6% [5]-[6]. However, scant information is available on the prevalence and characterization of animal-origin MRSA in India. A report on bovine mastitis by Kumar *et al*, 2011 indicated MRSA prevalence rate of 13.1% in cattle, as did another current report which showed a prevalence rate of 9.6% [6]-[7]. A lower prevalence of MRSA infections in cattle have been reported in the USA (4%) and Korea (4.2%), although a report from China showed a high prevalence of MRSA strains (47.6%) [8]—[10].

Treatment of mastitis in India is mainly dependent on antimicrobial therapy such β-lactams, cephalosporin, aminoglycoside, polyketide, macrolide, etc [11–13]. Infections caused by MRSA strains are resistant towards major classes of antibiotics which make the treatment prolonged, leaving veterinarians with limited options. Expression of the *mecA* gene in MRSA strains present on Staphylococcal cassette chromosome *mec* (SCC*mec*), a mobile genetic element results in decreased susceptibility to methicillin/oxacillin [14]. Numerous reports of MRSA strains causing infections in both humans and animals with a potential of zoonotic transmission are reported worldwide [15–19]. The emergence of oxacillin susceptible *mecA* positive *S*. *aureus* strains (OS-MRSA) has further complicated the scenario of diagnosis and treatment. The OS-MRSA strains have been reported in animal and human infections worldwide [10], [20–23], however, in India these strains were only reported in human infections [22]. The slow but increasing prevalence of OS-MRSA strains across the globe indicates that they may outnumber the existing MRSA strains in the near future and become a huge concern as they are not easily detectable [24].

Recently, a homologue of the *mecA* gene which cannot be amplified using *mecA* gene primers referred as *mecC*, has been identified in MRSA infections [19]. The occurrence of such *mecA* negative and *mecC* positive MRSA strains has further contributed to increasing diagnostic complexity. The other hurdles to the existing problem are the emergence of vancomycin resistant *S*. *aureus* (VRSA) and heterogeneous vancomycin intermediate strains (h-VISA), with decreased sensitivity to the antibiotic considered a drug of last resort for MRSA treatment in humans [25], [26]. VRSA strains have surfaced from different parts of the globe, one harboring the *vanA* gene conferring resistance phenotype transmitted by vancomycin resistant *Enterococci* (VRE) and the other devoid of it. VRE is the common pathogen in the farm animals, which makes it more crucial to investigate the presence of the *vanA* gene and determine vancomycin susceptibility in *S*. *aureus* isolates [27].

The prevailing phenotypic and genotypic variability of MRSA strains pose a threat to public health. Those at greatest risk are the farm caretakers, farm animals and veterinarians. Given the potential of zoonotic transmission, the surveillance of the antibiotic sensitivity profile, along with the genotype of the prevailing pathogenic strains, and their cross tolerance to other antibiotics is crucial. The sensitivity profile is important for devising new policies on therapeutic approaches. There are limited reports from India describing the variable traits of MRSA strains causing bovine mastitis using both phenotypic and genetic tests [6]-[7], [28]-[29]. A key focus of the study is to investigate the genetic and antibiotic susceptibility profiling of *S*. *aureus* isolates from the milk of cows suffering from mastitis in India.

## Methods

### Milk Sample Collection and Isolation of *S*. *aureus*

Milk samples (n = 167) were collected from cows showing symptoms of clinical mastitis (decreased milk production, change in milk color, redness and inflammation of the udder) by a professional veterinarian. Milk samples were collected from farms in three different states of India, Telangana (n = 78), Andhra Pradesh (n = 50) and Tamil Nadu (n = 39). Milk samples were obtained after cleaning the teats, followed by discarding first few streams of milk and then again cleaning the teats with cotton moistened with 70% alcohol.

*S*. *aureus* strains were isolated by adding the aliquots of milk samples to trypticase soy broth and incubating overnight at 37°C. The broth was then streaked on Baird Parker agar containing 30% egg yolk emulsion and 1% potassium tellurite and then onto mannitol salt agar plate. Yellow colored colonies on the mannitol salt agar plate were presumed to be *S*. *aureus*. Further, identification and characterization of the *S*. *aureus* colonies were done with the help of commercially available biochemical tests kit including catalase assay (BBL™ BD catalase reagent droppers,BD- 261203), Latex test (HiStaph™ Latex Test Kit- Himedia- LK03), Coagulase (Himedia- FD248) and Gram staining kit (Sigma Aldrich- 77730).

### PCR Amplification

To further confirm the identity of the biochemically-characterized strains genomic DNA was isolated using Wizard genomic kit (Promega). Briefly, 2mL of bacterial cultures in Mueller Hinton Agar grown overnight were pelleted at 10,000X g for 3 mins. The cell pellet was washed with 1X PBS twice at 8000X g, 3 mins and suspended in 500 μl of 50mM EDTA, lysostaphin (100μg/ml) and lysozyme (100μg/ml) for 1hr at 37°C prior to following the manufacturer’s instructions. Genomic DNA was used to perform PCR amplification of the following genes, *16S rRNA*, *mecA*, *mecC*, *vanA*, *pvl*, SCC*mec* typing, *agr* typing and *spa* typing [30–34]. PCR products of *16S rRNA* gene were sequenced and further confirmed by carrying out BLAST analysis.

### *Spa* Typing and Multilocus Sequence Typing (MLST)

The *spa* gene repeats were amplified from all the isolates using the standard primer and protocol available on Ridom Staph Type (www.ridom.com). The amplified PCR product was purified using MN PCR purification kit and sequenced. Further, the *spa* type was deduced by using the available online database (http://spatyper.fortinbras.us/).

MLST analysis was performed by sequencing internal fragments of seven housekeeping genes, Carbamate kinase (*arc*), Shikimate dehydrogenase (*aroE*), Glycerol kinase (*glpF*), Guanylate kinase (*gmk*), Phosphate acetyltransferase (*pta*), Triosephosphate isomerase (*tpi*), and Acetyl coenzyme A acetyltransferase (*yqiL*) using procedures described elsewhere (http://www.mlst.net/). Sequence types (STs) were assigned by comparison with the *S*. *aureus* MLST database (http://www.mlst.net/).

### Antibiotic Susceptibility (Disc Diffusion, Micro-Broth Dilution)

All isolates were tested for susceptibility to commonly used antibiotics by using two methods, i.e., disc diffusion assay and micro-broth dilution method as per CLSI guidelines [35]. The disc diffusion assay was performed with ampicillin (10μg), clindamycin (2 μg), erythromycin (15 μg), gentamycin (10 μg), ciprofloxacin (5 μg), tetracycline (30 μg), rifampicin (5 μg), cefoxitin (30 μg) and teicoplanin (30 μg). The resazurin dye based micro-broth dilution assay was used to determine MICs against oxacillin, vancomycin and linezolid as described previously [36]. ATCC 29213 (methicillin sensitive control strain) and ATCC 25923 (methicillin resistant control strain) were used as a control for disc diffusion and micro broth dilution assays. All assays were repeated at least twice in triplicates.

### Ethics Statement

Prior to collection of milk samples from infected cow’s oral consent was given by the farm care takers. There is no need to obtain any permission for milk sample collection from animal as per Indian law. Further, milk samples were collected by the professional veterinarians. The study does not involve any endangered species or protected species and no animals were used for any experiments.

## Results

### Antibiotic Susceptibility Profiling of *S*. *aureus* Isolates

39 isolates of *S*. *aureus* were obtained from milk samples and antibiotic susceptibility profiles to 12 antibiotics were determined as shown in Table 1. High resistance rates were observed towards clindamycin (76.92%), erythromycin (64.10%), ampicillin (56.41%), tetracycline (41.02%) and ciprofloxacin (35.89%) whereas low resistance was seen against rifampicin (20.51%) and gentamycin (15.38%). No resistance was found towards oxacillin, cefoxitin, vancomycin, teicoplanin and linezolid. Out of 39 strains, 24 isolates (62%) showed a multidrug resistant phenotype (resistant to 3 or more than 3 classes of antibiotics), followed by 13 strains (33%) with resistance towards one or two antibiotics and only 2 isolates (5%) were pan-sensitive. All isolates were susceptible to oxacillin, while 11 isolates (28.2%) showed reduced vancomycin susceptibility (MIC = 2μg/ml).

**Table 1:** Genotypic and phenotypic characterization of MRSA and MSSA clinical isolates causing bovine mastitis in India.

S.no	Isolate Id	Amp	Clin	Ery	Gen	Cip	Tet	Rif	Tei	Cef	Oxa MIC (μg/ml)	Van MIC (μg/ml)	Lin MIC (μg/ml)	*mecA*	SCC*mec* type	MLST*	*Spa* type	*Agr* type	*pvl*
1.	**TG-1**	R	R	R	R	S	R	S	S	S	<0.5	0.5	4	+	V	ST-1687	t7684	1	+
2.	**TG-23**	R	R	R	S	R	R	R	S	S	<0.5	1	2	+	NT	ST-2459	t267	1	+
3.	**TG-28**	R	R	R	R	R	S	S	S	S	1.25	2	2	+	V	ST-72	t7287	1	+
4.	**TG-29**	R	R	R	S	R	S	R	S	S	<0.5	1	2	+	V	ST-72	t7287	3	+
5.	**TG-36**	S	S	S	S	S	S	S	S	S	<0.5	1	2	+	III	ST-2459	t267	1	+
6.	**TG-52**	R	S	R	S	S	R	S	S	S	<0.5	2	2	+	IVc	ST-239	t037	1	+
7.	**TG-55**	S	S	S	S	R	S	S	S	S	<0.5	1	2	+	III+ IVc	ST-88	t2526	NT	+
8.	**TG-66**	R	R	R	S	S	S	R	S	S	1.25	0.5	2	+	III + IVc	ST-1687	t7696	1	+
9.	**TG-67**	R	R	R	S	S	S	R	S	S	<0.5	1	2	+	III + IVc	ST-1687	t7696	1	+
10	**TG-71**	R	S	R	S	R	R	S	S	S	0.63	1	2	+	IVc	ST-239	t037	1	+
11	**TG-72**	R	R	R	S	R	R	S	S	S	0.63	1	2	+	IVc	ST-239	t037	1	+
12	**TN-8**	R	R	R	S	S	S	S	S	S	<0.5	1	2	+	III	ST-1687	t2445	1	+
13	**TN-9**	R	R	R	S	R	S	S	S	S	<0.5	0.5	2	+	III	ST-1687	t7696	1	+
14	**TN-13**	S	R	R	S	S	S	S	S	S	<0.5	1	2	+	III	ST-63	t7287	3	+
15	**TN-38**	S	R	S	S	S	S	S	S	S	<0.5	1	2	+	III	ST-2459	t521	1	+
16	**AP-9**	R	R	R	R	R	R	S	S	S	<0.5	2	2	+	V	ST-72	t3731	1	+
17	**AP-20**	R	R	R	R	R	S	S	S	S	<0.5	2	2	+	V	ST-72	t8137	1	+
18	**AP-44**	R	R	S	R	S	S	R	S	S	0.5	2	2	+	NT	ST-72	t7286	1	-
19	**AP-49**	S	R	S	S	R	S	S	S	S	0.5	1	2	+	III	ST-72	t7286	1	-
20	**TG-33**	S	S	S	S	S	S	S	S	S	<0.5	1	4	-	-	ND	t267	1	+
21	**TG-50**	R	S	R	S	S	R	S	S	S	<0.5	2	2	-	_	ND	t037	1	+
22	**TG-64**	R	S	S	S	S	S	S	S	S	<0.5	1	2	-	_	ND	t2164	2	+
23	**TG-74**	R	S	R	S	S	R	S	S	S	0.63	1	2	-	_	ND	t037	1	+
24	**TN-1**	S	R	R	S	S	S	R	S	S	<0.5	1	2	-	_	ND	t7684	1	+
25	**TN-2**	S	R	S	S	S	R	S	S	S	<0.5	0.5	2	-	_	ND	t7684	NT	-
26	**TN-3**	R	R	R	S	S	R	R	S	S	<0.5	2	2	-	_	ND	t2445	3	+
27	**TN-7**	S	S	R	S	S	S	S	S	S	<0.5	0.5	2	-	_	ND	t7684	3	-
28	**TN-10**	R	R	R	S	R	S	S	S	S	<0.5	0.5	2	-	_	ND	t7696	1	+
29	**TN-15**	S	R	S	S	S	S	S	S	S	<0.5	1	2	-	_	ND	t7287	3	+
30	**TN-16**	R	R	R	S	R	S	S	S	S	0.5	2	2	-	_	ND	t2445	1	+
31	**TN-19**	S	R	S	S	S	R	S	S	S	<0.5	1	2	-	_	ND	t9602	1	+
32	**TN-20**	R	R	R	R	S	R	R	S	S	<0.5	1	2	-	_	ND	t521	1	+
33	**TN-21**	S	R	R	S	R	R	S	S	S	<0.5	0.5	2	-	_	ND	t2246	1	+
34	**TN-27**	R	R	R	S	R	S	S	S	S	<0.5	0.5	2	-	_	ND	t2246	3	+
35	**TN-39**	S	R	S	S	S	S	S	S	S	<0.5	1	2	-	_	ND	t605	1	+
36	**AP-8**	S	R	S	S	S	R	S	S	S	<0.5	2	2	-	_	ND	t7286	2	-
37	**AP-23**	S	R	S	S	S	R	S	S	S	<0.5	2	2	-	_	ND	t7286	2	-
38	**AP-43**	S	R	S	S	S	S	S	S	S	<0.5	1	2	-	_	ND	t7287	3	+
39	**AP-45**	S	R	R	S	S	R	S	S	S	<0.5	2	2	-	_	ND	t7286	2	-

TG, TN and AP refer to strains isolated from Telangana, Tamil Nadu and Andhra Pradesh. The disc diffusion assay was performed with Amp (ampicillin, 10μg), Clin (clindamycin, 2 μg), Ery (erythromycin, 15 μg), Gen (gentamycin,10 μg), Cip (ciprofloxacin, 5μg), Tet (tetracycline, 30 μg), Rif (rifampicin,5 μg), Cef (cefoxitin, 30 μg) and Tei (teicoplanin, 30 μg). S denotes Sensitive and R denotes resistant strains against disc diffusion assay. Micro-broth dilution assays were performed for three drugs: Oxacillin (Oxa), Vancomycin (Van) and Linezolid (Lin). NT denotes non-typeable strains. **+** indicates positive for the gene and–denotes negative for the gene. No SCC*mec* typing was performed for *mecA* negative strains.

* MLST was only performed for MRSA clones, ND- Not determined.

### Presence of Resistance Determinants and SCC*mec* Element Classification

Only 19 isolates were found to be positive for the *mecA* gene (determinant of methicillin resistance) while all were negative for the *mecC* gene (homologue of *mecA* gene). The *mecA* positive isolates belonged to SCC*mec* Class Type III (n = 6), Type IVc (n = 3), Type V (n = 5), while 3 strains showed mixed type (Type III+IVc) and 2 strains were non-typeable. All strains were negative for the *vanA* gene, responsible for vancomycin resistance.

### Molecular Typing and *Pvl* Gene Amplification

The majority of the strains belonged to *agr* group I (66.7%), followed by *agr* group II (10.3%) and group III (17.9%). The strains belonged to variable *spa* types, t037 (n = 5), t267 (n = 3), t521 (n = 2), t605 (n = 1), t2164 (n = 1), t2246 (n = 2), t2445 (n = 3), t2526 (n = 1), t3731 (n = 1), t7286 (n = 5), t7287 (n = 5), t7684 (n = 4), t7696 (n = 4), t8137(n = 1), and t9602 (n = 1). Furthermore, 32 isolates were found to be positive for the *pvl* gene.

### Characterization of OS-MRSA Strains

Antibiotic sensitivity revealed all strains to be oxacillin sensitive, however, 19 isolates (48.7%) were positive for the *mecA* gene and so represented the OS-MRSA population. Of these 19 OS-MRSA strains, 14 were multidrug resistant, 4 were resistant to one or two antibiotics and there was a single pan-sensitive strain. The majority of OS-MRSA strains (n = 17) were *pvl* positive and most belonged to *agr* group 1 (n = 16) followed by *agr* type III (n = 2). One isolate was non-typeble. Genetic diversity was observed among OS-MRSA strains with MLST sequence types, ST72 (n = 6), ST-2459 (n = 3), ST-239 (n = 3), ST-88 (n = 1), ST-63 (n = 1) and five strains with novel ST-1687 Table 1. All isolates belonged to different SCC*mec* class and *spa* types showing clonal variation. OS-MRSA strains were found in all farms with 11 OS-MRSA strains found in Telangana, followed by 4 strains each found in Tamil Nadu and Andhra Pradesh.

## Discussion

In this study, the genotypes and phenotypes of pathogenic strains of *S*. *aureus* from three different states of India were characterized. Among the isolates obtained 48.71% were OS-MRSA strains and to the best of our knowledge, they are reported for the first time in animal infections from India. The occurrence of OS-MRSA strains detected in the present study, in conjunction with reports from other parts of world, accentuates the need to include genetic tests along with phenotypic tests for accurate representation of the prevailing strains in the population [10], [21]-[24]. Using only phenotypic tests could result in misidentification of OS-MRSA as methicillin susceptible *S*. *aureus* strains (MSSA), this will misrepresent of the prevalence of OS-MRSA and further complicate treatment. The actual prevalence of OS-MRSA may be much higher in bovine mastitis cases than currently reported in the literature, as these strains would be missed by antibiotic susceptibility assays.

OS-MRSA strains were found in all the three states sampled, with the majority of them testing as multidrug resistant, however, all were sensitive to teicoplanin, vancomycin and linezolid. Further, molecular typing of the isolates revealed that they belonged to different SCC*mec*, *agr*, *spa* and MLST type. *spa* typing results indicated all strains were genotypically variable from the different areas investigated. This is in contrast to a previous study that showed OS-MRSA strains from a farm in Gansu province of China belonged to single parental lineage (t267 *spa* type). In the same study OS-MRSA from other farms belonged to two *spa* types, t1234 and t267 [10]. Further, reports of MRSA from Korea showed that 11 out of 17 mastitic strains from five different farms belonged to a single *spa* type (t324) and report from the USA found that all strains (n = 4) belonged to similar *spa* (t121) type [8]-[9]. Among the strain types that we found, *spa* types t2526 and t9602 have been previously reported in human infections [37]-[38]. Other *spa* types were well documented in both human and bovine infections or in bovine mastitis except *spa* types, t3731 and t8137 for which no published reports could be found.

MLST analysis of the OS-MRSA strains revealed diversity, however, all the four strains from Andhra Pradesh belonged only to ST-72. A novel sequence type, ST-1687 was also found among the strains from Telangana and Tamil Nadu. Notably, other major MRSA clones causing bovine mastitis reported worldwide from countries like, China (ST-97, ST-9, ST-71 and ST-2738), Belgium (ST-398 and ST-8), Switzerland (ST-398) and USA (ST-8 and ST-5) were not found [8], [39–43]. We have also found strains belonging to ST-239 and ST-72 lineages which were reported from cases of human and animal infections [9], [44]-[45]. A strain with ST-88 lineage was identified, which is a major human MRSA clone from Africa and also has been reported in Asia, Europe and South America [20]. Interestingly, a recent report by Conceicao et al., 2015, also showed ST-88 as a major prevalent OS-MRSA clone causing human infections [20]. Therefore, the findings of the strains with MLST and *spa* type known in human infection in the current study may point towards transmission between human to animals. We also found *pvl* positive isolates, which are rarely reported in animal origin strains [8]-[10]. The *pvl* gene is also a virulence factor that is mainly associated with community acquired-MRSA (CA-MRSA) infections that causes serious pathology including skin infections and necrotizing pneumonia in humans [46–48]. The high prevalence of *pvl* positive strains along with human specific lineages raises the concerning possibility of human to animal transfer or vice versa. The presence of the *pvl* gene in OS-MRSA strains suggest that these strains may be highly virulent and further in lack of effective treatment regimen may result in non-curable and fatal mastitic infections.

Keeping in mind, the reported prevalence of OS-MRSA strains from different parts of the globe, studies providing in depth insight into the inexplicable resistance mechanism and their fitness levels are of the utmost importance. These studies will help to genetically characterize the OS-MRSA strain from MRSA and so provide valuable information.

## Conclusion

This study reports genotypically variable strains causing bovine mastitis in cows belong to different lineages, and strains linked to lineages known to cause human infections are present in India. Overall, the results indicate the need to include genetic testing along with phenotypic testing to prevent the misinterpretation of OS-MRSA as MSSA and also the need for studies to better understand this mysterious OS-MRSA phenotype.
