# Correction: Prevalence and Characterization of Oxacillin Susceptible *mecA*-Positive Clinical Isolates of *Staphylococcus aureus* Causing Bovine Mastitis in India

**DOI:** 10.1371/journal.pone.0232348

**Published:** 2020-04-22

**Authors:** Hiral Mistry, Paresh Sharma, Sudipta Mahato, R. Saravanan, P. Anand Kumar, Vasundhra Bhandari

The authors incorrectly denoted ATCC 25923 as a methicillin resistant control strain in the penultimate sentence of the Antibiotic Susceptibility (Disc Diffusion, Micro-Broth Dilution) subsection of the Methods. The correct sentence is: ATCC 29213 (methicillin sensitive control strain) and ATCC 25923 (a strain used as a control in some antibiotic susceptibility testing, not methicillin resistant) were used as a control for micro broth dilution and disk diffusion assay, respectively.

The authors would like to acknowledge the importance of using a genuinely positive MRSA control strain in phenotypic testing and interpreting results.

There are errors in Table 1. The authors have amended the data to correct their dilution series and reflect the actual concentrations used. The authors apologize for the errors in the table. Please see the correct Table 1 here.

**Table 1:** Genotypic and phenotypic characterization of MRSA and MSSA clinical isolates causing bovine mastitis in India.

S.no	Isolate Id	Amp	Clin	Ery	Gen	Cip	Tet	Rif	Tei	Cef	Oxa MIC (μg/ml)	Van MIC (μg/ml)	Lin MIC (μg/ml)	*mecA*	SCC*mec* type	Spa typing	Agr type	pvl
1.	**TG-1**	R	R	R	R	S	R	S	S	S	0.312	0.5	4	+	V	t7684	1	+
2.	**TG-23**	R	R	R	S	R	R	R	S	S	0.312	1	2	+	NT	t267	1	+
3.	**TG-28**	R	R	R	R	R	S	S	S	S	1.25	2	2	+	V	t7287	1	+
4.	**TG-29**	R	R	R	S	R	S	R	S	S	0.312	1	2	+	V	t7287	3	+
5.	**TG-36**	S	S	S	S	S	S	S	S	S	0.156	1	2	+	III	t267	1	+
6.	**TG-52**	R	S	R	S	S	R	S	S	S	0.312	2	2	+	IVc	t037	1	+
7.	**TG-55**	S	S	S	S	R	S	S	S	S	0.156	1	2	+	III+ IVc	t2526	NT	+
8.	**TG-66**	R	R	R	S	S	S	R	S	S	1.25	0.5	2	+	III + IVc	t7696	1	+
9.	**TG-67**	R	R	R	S	S	S	R	S	S	0.312	1	2	+	III + IVc	t7696	1	+
10.	**TG-71**	R	S	R	S	R	R	S	S	S	0.625	1	2	+	IVc	t037	1	+
11.	**TG-72**	R	R	R	S	R	R	S	S	S	0.625	1	2	+	IVc	t037	1	+
12.	**TN-8**	R	R	R	S	S	S	S	S	S	0.312	1	2	+	III	t2445	1	+
13.	**TN-9**	R	R	R	S	R	S	S	S	S	0.312	0.5	2	+	III	t7696	1	+
14.	**TN-13**	S	R	R	S	S	S	S	S	S	0.156	1	2	+	III	t7287	3	+
15.	**TN-38**	S	R	S	S	S	S	S	S	S	0.156	1	2	+	III	t521	1	+
16.	**AP-9**	R	R	R	R	R	R	S	S	S	0.312	2	2	+	V	t3731	1	+
17.	**AP-20**	R	R	R	R	R	S	S	S	S	0.156	2	2	+	V	t8137	1	+
18.	**AP-44**	R	R	S	R	S	S	R	S	S	0.312	2	2	+	NT	t7286	1	-
19.	**AP-49**	S	R	S	S	R	S	S	S	S	0.312	1	2	+	III	t7286	1	-
20.	**TG-33**	S	S	S	S	S	S	S	S	S	0.312	1	4	-	-	t267	1	+
21.	**TG-50 **	R	S	R	S	S	R	S	S	S	0.156	2	2	-	_	t037	1	+
22.	**TG-64**	R	S	S	S	S	S	S	S	S	0.312	1	2	-	_	t2164	2	+
23.	**TG-74**	R	S	R	S	S	R	S	S	S	0.625	1	2	-	_	t037	1	+
24.	**TN-1**	S	R	R	S	S	S	R	S	S	0.312	1	2	-	_	t7684	1	+
25.	**TN-2**	S	R	S	S	S	R	S	S	S	0.312	0.5	2	-	_	t7684	_	-
26.	**TN-3**	R	R	R	S	S	R	R	S	S	0.312	2	2	-	_	t2445	3	+
27.	**TN-7**	S	S	R	S	S	S	S	S	S	0.156	0.5	2	-	_	t7684	3	-
28.	**TN-10**	R	R	R	S	R	S	S	S	S	0.156	0.5	2	-	_	t7696	1	+
29.	**TN-15**	S	R	S	S	S	S	S	S	S	0.156	1	2	-	_	t7287	3	+
30.	**TN-16**	R	R	R	S	R	S	S	S	S	0.156	2	2	-	_	t2445	1	+
31.	**TN-19**	S	R	S	S	S	R	S	S	S	0.312	1	2	-	_	t9602	1	+
32.	**TN-20**	R	R	R	R	S	R	R	S	S	0.312	1	2	-	_	t521	1	+
33.	**TN-21**	S	R	R	S	R	R	S	S	S	0.156	0.5	2	-	_	t2246	1	+
34.	**TN- 27**	R	R	R	S	R	S	S	S	S	0.312	0.5	2	-	_	t2246	3	+
35.	**TN-39**	S	R	S	S	S	S	S	S	S	0.312	1	2	-	_	t605	1	+
36.	**AP-8**	S	R	S	S	S	R	S	S	S	0.156	2	2	-	_	t7286	2	-
37.	**AP-23**	S	R	S	S	S	R	S	S	S	0.312	2	2	-	_	t7286	2	-
38.	**AP-43**	S	R	S	S	S	S	S	S	S	0.156	1	2	-	_	t7287	3	+
39.	**AP-45**	S	R	R	S	S	R	S	S	S	0.156	2	2	-	_	t7286	2	-

TG, TN and AP refer to strains isolated from Telangana, Tamil Nadu and Andhra Pradesh. The disc diffusion assay was performed with Amp (ampicillin, 10μg), Clin (clindamycin, 2 μg), Ery (erythromycin, 15 μg), Gen (gentamycin,10 μg), Cip (ciprofloxacin, 5μg), Tet (tetracycline, 30 μg), Rif (rifampicin,5 μg), Cef (cefoxitin) and Tei (teicoplanin, 30 μg). S denotes Sensitive and R denotes resistant strains against disc diffusion assay. Micro-broth dilution assay was carried out for Oxa denotes oxacillin; Van denotes vancomycin and Lin denotes linezolid drug. NT denotes non-typeable strains. + indicates positive for the gene and–denotes negative for the gene. No *mec* typing was performed for *mecA* negative strains.
